# Design of Cu_2_O_(O)_@Cu_2_O_(P)_@AuPt Multilevel Core–Shell Heterostructures via Mild Reduction Strategy with a Dual Function for Efficient Photocatalytic Degradation

**DOI:** 10.3390/ma19143069

**Published:** 2026-07-16

**Authors:** Bo Ma, Guoqiang Huang, Wenwen Hu, Wenxue An, Gailan Ma, Maohui Li, Youjun Lu

**Affiliations:** 1 School of Materials Science & Engineering, North Minzu University, Yinchuan 750021, China; 2 Institute of Semiconductor Crystals and Ceramic Materials, Helanshan Laboratory, Yinchuan 750021, China; 3 National and Local Joint Engineering Research Center of Advanced Carbon-Based Ceramics Preparation Technology, Yinchuan 750021, China

**Keywords:** photocatalyst, metal semiconductor, core–shell, alloy, cuprous oxide

## Abstract

The degradation of organic pollutants through photocatalysis is currently a major research focus. Core–shell heterostructures of metal semiconductors have been widely recognized as an effective strategy for enhancing photocatalytic performance, particularly when alloy nanoparticles are incorporated due to their unique electronic and catalytic properties. However, conventional synthetic approaches typically rely on high-temperature and high-pressure conditions, which often induce undesirable particle overgrowth and aggregation. Herein, AuPt bimetallic alloy nanoparticles were successfully fabricated via two successive in situ redox processes under room-temperature and ambient-pressure conditions, which were in situ integrated with Cu_2_O to form multilevel core–shell composite particles. Structurally, an octahedral Cu_2_O crystal serves as the inner core (denoted as Cu_2_O_(O)_), sequentially coated with a Cu_2_O nanoparticle (denoted as Cu_2_O_(P)_) interlayer and a AuPt alloy nanoparticle shell. Functionally, the enhanced photocatalytic activity of Cu_2_O_(O)_@Cu_2_O_(P)_@AuPt was proven to be attributed to a dual function of AuPt, which includes an adsorption-induced polarized interface and an efficient charge-transfer mediator with the ohmic contact. This work demonstrates a mild and versatile synthetic strategy for constructing semiconductor–alloy heterostructures, offering valuable insights into the rational design of highly efficient and stable photocatalysts.

## 1. Introduction

Semiconductor-based photocatalysis has emerged as a promising and sustainable strategy for addressing critical environmental challenges, including organic pollutant degradation and solar-to-chemical energy conversion [1,2,3,4]. Tetracycline (TC) and methyl orange (MO) were deliberately chosen as model organic pollutants to cover two major classes of refractory water contaminants: pharmaceutical antibiotics and industrial azo dyes. TC residues readily induce the spread of antibiotic-resistant genes, and MO is difficult to mineralize and generates toxic intermediates under natural water conditions. Among the various semiconductor materials, a typical p-type semiconductor of cuprous oxide (Cu_2_O) has attracted considerable attention due to the strong visible-light absorption, low cost, earth abundance, and favorable band structure for photocatalytic applications [5,6,7,8]. However, despite these merits, pristine Cu_2_O suffers from several intrinsic limitations that severely restrict its practical photocatalytic performance [9,10,11]. One of the primary challenges lies in the rapid recombination of photogenerated electron–hole pairs, which significantly reduces quantum efficiency [12,13,14]. More critically, Cu_2_O is thermodynamically unstable under illumination conditions and is highly susceptible to photocorrosion [15,16,17,18,19]. This instability not only deteriorates catalytic performance but also limits long-term usability. To overcome these issues, extensive research efforts have been devoted to engineering heterostructured systems, which can be recognized as an effective strategy to simultaneously address both carrier recombination and photocorrosion [20,21,22].

Among various approaches, integrating metallic components with semiconductors to form metal-semiconductor heterojunctions has proven highly effective [23]. The introduction of metals can induce the formation of ohmic contact and Schottky junctions at the interface, which facilitate directional charge transfer and suppress electron–hole recombination [24]. In this context, alloy nanoparticles have garnered increasing interest due to their unique electronic structures arising from synergistic interactions between constituent metals [25]. Compared with monometallic counterparts, alloy systems exhibit tunable d-band centers and modified Fermi levels, which can significantly influence adsorption behavior and charge-transfer dynamics [26,27]. These characteristics often result in superior photocatalytic performance, particularly in reactions involving complex molecular transformations [28,29]. Recent studies have demonstrated that loading alloy nanoparticles such as Au-Pt, Pd-Cu, or Pt-Ni onto semiconductor substrates can effectively enhance photocatalytic activity [30,31]. These systems typically benefit from improved charge separation efficiency and enhanced surface reaction kinetics [32,33].

However, in most reported cases, the alloy nanoparticles are synthesized under high-temperature or high-pressure conditions before being combined with Cu_2_O [31,34,35]. Such harsh synthesis environments often lead to undesirable effects, including particle coarsening and severe aggregation [36,37]. More importantly, the contradictory synthetic requirements between alloy formation and Cu_2_O structural preservation greatly restrict the facile and controllable preparation of this composite material. Extreme thermal and pressure conditions easily trigger the oxidation of monovalent copper in Cu_2_O and induce unwanted phase transformation, destroying the intrinsic structure of the Cu_2_O matrix [38,39]. Therefore, developing mild and controllable synthetic strategies that enable the formation of alloy–Cu_2_O heterostructures under ambient conditions remains a significant scientific challenge [40,41].

Herein, by leveraging the similar reduction potentials of Au and Pt precursors, we successfully immobilized AuPt alloy nanoparticles onto Cu_2_O to construct multilevel core–shell-structured particles under ambient temperature and atmospheric pressure. The architecture of Cu_2_O_(O)_@Cu_2_O_(P)_@AuPt (the original octahedral Cu_2_O framework in the inner region was denoted as Cu_2_O_(O)_, and the newly generated particulate Cu_2_O in the outer layer was denoted as Cu_2_O_(P)_) provides multiple functional heterointerfaces to synergistically boost photocatalytic activity. Furthermore, theoretical simulations reveal that the AuPt alloy surface plays a crucial role in facilitating the activation and cleavage of chemical bonds in adsorbed methyl orange molecules. This approach is expected to be broadly applicable to other material systems and provides valuable insights into interface engineering, alloy design, and the development of highly efficient and stable photocatalysts for environmental and energy applications.

## 2. Experimental Section

### 2.1. Materials

All chemicals were of analytical grade and used as received without further purification. Copper(II) sulfate pentahydrate (CuSO_4_·5H_2_O), sodium hydroxide (NaOH), glucose (C_6_H_12_O_6_), hydroquinone (C_6_H_6_O_2_), polyvinyl–pyrrolidone (PVP), chloroplatinic acid (H_2_PtCl_6_·6H_2_O), chloroauric acid (HAuCl_4_·4H_2_O), tetracycline (TC) and methyl orange (MO) were purchased from Aladdin Reagent Co., Ltd. (Shanghai, China). Deionized water was used throughout all experiments.

### 2.2. Synthesis of Cu_2_O Particles

Cu_2_O particles were synthesized via a wet-chemical reduction method. Typically, 0.4996 g of CuSO_4_·5H_2_O was dissolved in 30 mL of deionized water to form a clear blue copper sulfate solution. Separately, 6.8 g of NaOH was dissolved in 25 mL of deionized water. Then, the CuSO_4_·5H_2_O solution was heated to 55 °C in a water bath under continuous magnetic stirring, after which the NaOH solution was added dropwise. After 5 min, 0.5 g of C_6_H_6_O_2_ was introduced as the reductant. The reaction mixture was maintained for 30 min, and the obtained brick-red precipitates were collected by centrifugation, washed, and dried for further use.

### 2.3. Synthesis of Cu_2_O@Cu Particles

Cu_2_O@Cu particles were prepared by partial reduction of the pre-synthesized Cu_2_O. Specifically, 20 mg of Cu_2_O powder was dispersed in 30 mL of ethylene glycol by ultrasonication for 10 min to obtain a homogeneous suspension. Meanwhile, 2 g of NaOH was dissolved in 10 mL of deionized water, and 1.96 g of glucose was dissolved in another 10 mL of deionized water. The Cu_2_O suspension was heated to 60 °C in a water bath under magnetic stirring. Subsequently, the NaOH solution was added dropwise, and after 5 min, the glucose solution was introduced into the reaction system. The reaction was allowed to proceed for 40 min. The obtained precipitates were collected by centrifugation, washed, and dried for further use.

### 2.4. Synthesis of Cu_2_O_(o)_@Cu_2_O_(p)_@Aupt Particles

In a typical synthesis, 10 mg of Cu_2_O@Cu powder was dispersed in 10 mL of deionized water by ultrasonication. Then, 50 mg of PVP was added as a stabilizing agent, and the mixture was further ultrasonicated to ensure uniform dispersion. The resulting suspension was transferred to a magnetic stirrer and reacted with 1.25 mL of HAuCl_4_ (5 mM) and 0.75 mL of H_2_PtCl_6_ (5 mM) solutions. After stirring for 30 min at room temperature, the final product was collected by centrifugation, washed, and dried for further use.

### 2.5. Characterizations

The morphology and microstructure of the as-prepared samples were characterized by field-emission scanning electron microscopy (FESEM) and transmission electron microscopy (TEM). The crystalline phases were identified by X-ray diffraction analysis (XRD) and Fourier-transform infrared spectroscopy (FTIR). The elemental composition and spatial distribution were characterized by energy-dispersive spectrometry (EDS) elemental mapping. The surface chemical states were investigated by X-ray photoelectron spectroscopy (XPS), and the valence band positions were determined by valence-band XPS. The optical absorption properties were examined by UV–visible spectra. Photoluminescence (PL) spectroscopy, transient photocurrent response, and electrochemical impedance spectroscopy (EIS) were employed to evaluate the separation and transfer behavior of photogenerated charge carriers. The specific surface area and pore-size distribution were measured using the Brunauer–Emmett–Teller (BET) method. Electron paramagnetic resonance (EPR) spectroscopy was performed to detect the reactive radical species generated during photocatalysis. Detailed information on the instruments and operating conditions is provided in the Supplementary Data.

### 2.6. Photocatalytic Performance

The photocatalytic activity of the samples was evaluated by the degradation of TC and MO under visible-light irradiation. In a typical experiment, 10 mg of photocatalyst was dispersed in 50 mL of pollutant solution (20 mg/L or 50 mg/L). Prior to irradiation, the suspension was magnetically stirred in the dark for 30 min to establish adsorption–desorption equilibrium between the catalyst surface and pollutant molecules. Subsequently, the suspension was irradiated using a 300 W Xe lamp (Perfectlight Technology Co., Ltd., Beijing, China) equipped with a 420 nm cut-off filter. At given time intervals, approximately 3 mL of the suspension was withdrawn, centrifuged, and filtered to remove solid particles. The residual concentration of the pollutant was analyzed using a UV-vis spectrophotometer (Hitachi, Ltd., Tokyo, Japan) by monitoring the characteristic absorption peak.

## 3. Results and Discussion

### 3.1. Structure and Component

**Figure 1:**
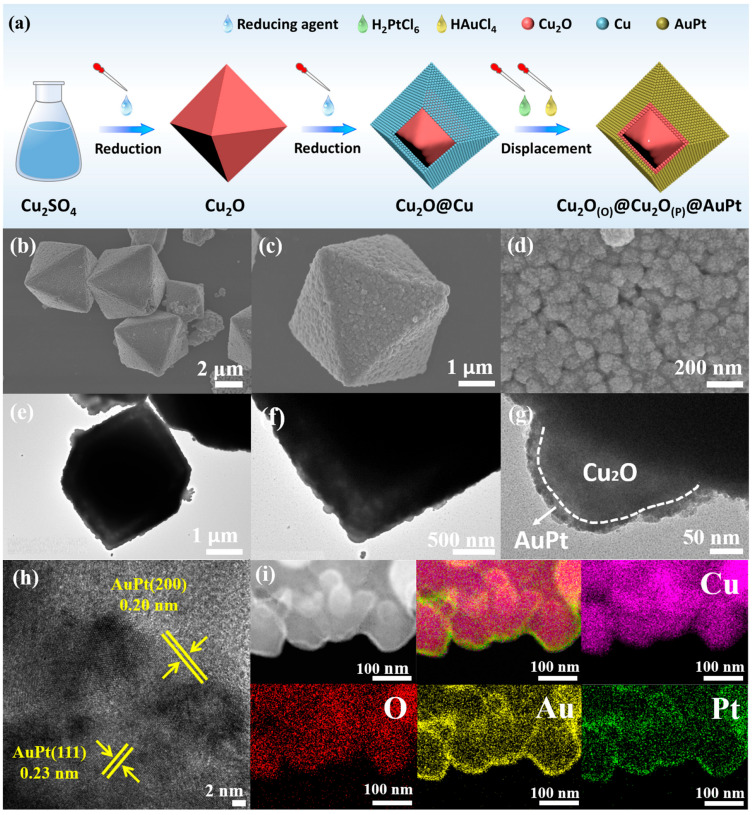
(**a**) Schematic diagram of the synthesis of samples. The images of Cu_2_O_(O)_@Cu_2_O_(P)_@AuPt for (**b**–**d**) SEM, (**e**–**g**) TEM, (**h**) HRTEM, and (**i**) HAADF-STEM and the corresponding EDS mapping.

Figure 1a schematically illustrates the synthetic route toward the Cu_2_O_(O)_@Cu_2_O_(P)_@AuPt core–shell–shell heterostructure. First, well-defined octahedral Cu_2_O particles were synthesized and served as sacrificial templates for the subsequent surface reconstruction process. Under alkaline reducing conditions, part of the Cu^+^ species in Cu_2_O was further reduced to metallic Cu, leading to the nucleation of Cu nanoparticles on the surface of the octahedral Cu_2_O. With prolonged reaction time, the surface Cu nanoparticles gradually grew and became densely packed, eventually forming a compact Cu shell around the Cu_2_O core, thus yielding Cu_2_O@Cu particles [42]. Subsequently, after the simultaneous introduction of HAuCl_4_ and H_2_PtCl_6_, a galvanic replacement reaction occurred at the Cu shell because metallic Cu is more readily oxidized than noble-metal ions are reduced. However, from a mechanistic standpoint, one of the key challenges in ambient-condition synthesis lies in the mismatch of reduction kinetics between different metal precursors [31]. Differences in standard reduction potentials often lead to sequential rather than simultaneous reduction, resulting in phase segregation or core–shell structures instead of homogeneous alloys. Therefore, selecting metal systems with closely matched reduction potentials represents a rational approach to achieving controlled alloy formation under mild conditions [43]. This concept provides an opportunity to design novel heterostructures with well-defined architectures and optimized electronic properties. For the Au-Pt system, the standard reduction potentials of Au^3+^/Au (+1.4 V) and Pt^2+^/Pt (+1.2 V) exhibit a relatively narrow gap compared with other bimetallic combinations, making the room-temperature, atmospheric-pressure wet co-reduction strategy thermodynamically feasible for fabricating uniform AuPt alloys. Therefore, Cu was oxidized and converted into the newly formed outer Cu_2_O_(P)_ shell, and precursors of precious metal ions were reduced in situ to AuPt alloys. The specific reaction chemical formulas can be found in (S1)–(S3) of the Supplementary Materials. Through this sequential reduction–reconstruction–replacement process, a unique Cu_2_O_(O)_@Cu_2_O_(P)_@AuPt hierarchical heterostructure with a multilevel core–shell structure was successfully constructed.

The as-prepared samples were further investigated in terms of their morphology and internal microstructure. As shown in Figure S1, the octahedral Cu_2_O particles exhibit a well-defined octahedral morphology with smooth surfaces and no obvious impurities, indicating the high uniformity of the synthesized product. The TEM image reveals a clear lattice fringe spacing of 0.300 nm, which can be assigned to the (110) plane of Cu_2_O, confirming the high crystallinity of the octahedral Cu_2_O. Moreover, the EDS elemental mapping images show that Cu and O are uniformly distributed throughout the particles, further verifying the homogeneous composition.

As shown in Figure S2, the obtained Cu_2_O@Cu particles largely retain the original octahedral morphology, suggesting that the template structure remains intact during the surface reduction process. However, in contrast to the smooth surface of pristine Cu_2_O, the outer surface of Cu_2_O@Cu becomes rough and is densely covered with numerous small particles with an average size of approximately 100 nm, indicating the formation of a particulate shell. The TEM image further reveals obvious interfacial voids between the inner core and the outer shell, demonstrating the formation of a distinct core–shell structure. Additionally, a lattice fringe spacing of 0.209 nm is observed, which matches well with the (111) plane of metallic Cu, confirming that the shell is composed of Cu nanoparticles. In addition, the EDS elemental mapping results show that the particle surface is dominated by Cu with only a minor O signal, further supporting the finding that the shell layer mainly consists of metallic Cu nanoparticles rather than copper oxide.

Figure 1b–i present the detailed morphology and microstructural features of the as-fabricated Cu_2_O_(O)_@Cu_2_O_(P)_@AuPt particles. As observed from the SEM images in Figure 1b–d, the final product largely preserves the overall octahedral morphology of the original template, indicating that the structural framework remains stable during the galvanic replacement process. Compared with Cu_2_O@Cu, the surface becomes significantly rougher. In particular, numerous ultrafine nanoparticles with an average size of approximately 5 nm are uniformly anchored on the larger shell particles, suggesting the successful deposition of noble-metal nanodomains on the reconstructed shell. As shown in Figure 1e–h, distinct voids can still be observed between the central core region and the outer shell, revealing that the product inherits the characteristic core–shell configuration of the intermediate Cu_2_O@Cu precursor. More importantly, the high-magnification TEM images of the shell clearly show that each shell particle possesses a composite structure: the inner part consists of Cu_2_O nanoparticles generated through oxidation of metallic Cu, whereas the outermost layer is decorated with ultrafine AuPt nanoparticles. In the HRTEM image, lattice fringes with spacings of 0.23 nm and 0.20 nm are observed, which can be assigned to the (111) and (200) planes of AuPt, respectively, confirming the presence of bimetallic AuPt nanocrystals on the shell surface. Furthermore, the elemental mapping results in Figure 1i and Figure S3 reveal a clear spatial compositional variation within each shell particle. Specifically, the inner region is mainly enriched in Cu and O, corresponding to Cu_2_O, while the outer region is dominated by Au and Pt, consistent with the formation of an AuPt outer layer. These results provide compelling evidence that the shell particles are reconstructed into a new Cu_2_O_(P)_@AuPt core–shell substructure. Therefore, the overall product can be reasonably described as a unique multilevel core–shell–shell heterostructure. The formation of such a hierarchical multilevel heterointerface is expected to promote interfacial charge separation and provide abundant surface-active sites, thereby benefiting the visible-light photocatalytic performance.

Figure S4 presents the XRD patterns of the three Cu_2_O-based samples. For all samples, the main diffraction peaks located at 36.42°, 42.30°, 61.34°, and 73.53° can be well indexed to the (111), (200), (220), and (311) planes of Cu_2_O, respectively, confirming that Cu_2_O is the dominant crystalline phase in these materials. In the case of Cu_2_O@Cu, three additional diffraction peaks appear at 43.30°, 50.43°, and 74.13°, which are assigned to the (111), (200), and (220) planes of metallic Cu, respectively. This result clearly demonstrates the successful formation of Cu nanoparticles on the Cu_2_O surface during the reduction process. For the final Cu_2_O_(O)_@Cu_2_O_(P)_@AuPt sample, only a very weak diffraction peak is observed at around 40.21°. This weak signal can be attributed to the low loading amount and ultrafine size of the AuPt nanoparticles, which results in poor diffraction intensity. More importantly, except for the characteristic peaks of Cu_2_O, no obvious diffraction peaks corresponding to metallic Cu can be detected in the final product. This observation indicates that the Cu shell in Cu_2_O@Cu was largely consumed during the galvanic replacement process and converted into newly formed Cu_2_O, which is in good agreement with the TEM results.

The FTIR spectra of the three samples are shown in Figure S5. All samples exhibit characteristic absorption bands associated with the Cu-O vibration, confirming the existence of Cu_2_O-related chemical bonds. Notably, no significant shift of the Cu-O band is observed among the three samples, suggesting that no new bulk copper-containing compounds were formed. In addition, the Cu_2_O sample shows the strongest Cu-O absorption intensity, whereas the corresponding bands of Cu_2_O@Cu and Cu_2_O_(O)_@Cu_2_O_(P)_@AuPt are relatively weaker. This difference can be reasonably attributed to the surface coverage of metallic Cu or AuPt-modified shell layers, which partially attenuates the infrared response of Cu-O bonds and is consistent with the observed morphological evolution of the samples.

**Figure 2:**
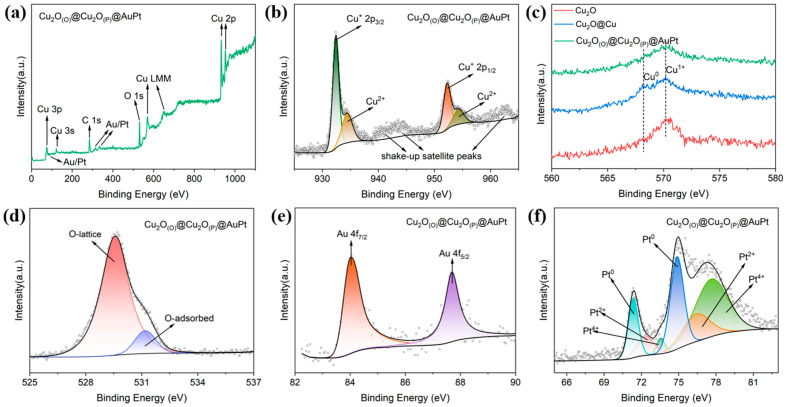
(**a**) XPS full spectrum of Cu_2_O_(O)_@Cu_2_O_(P)_@AuPt; (**b**) Cu 2p fine spectrum; (**c**) Cu LMM fine spectrum; (**d**) O 1s fine spectrum; (**e**) Au 4f fine spectrum; (**f**) Pt 4f fine spectrum.

To further elucidate the surface chemical composition and valence states of the as-prepared samples, XPS measurements were carried out in Figure 2 and Figure S6. First, the survey spectra of the three samples display prominent signals of Cu and O, confirming that these two elements are the main constituents of the materials [44]. For the Cu_2_O_(O)_@Cu_2_O_(P)_@AuPt sample (Figure 2a), weak but discernible signals of Au and Pt are also observed, indicating the successful deposition of AuPt species on the particle surface. The relatively low intensity of these peaks can be attributed to the low loading amount of the noble metals. Second, the Cu 2p spectra (Figure S6b) show two major peaks centered at approximately 932.6 eV and 952.2 eV, corresponding to Cu 2p_3/2_ and Cu 2p_1/2_ of Cu^+^ species, respectively. For Cu_2_O@Cu (Figure S6e), additional contributions associated with metallic Cu^0^ can be identified, confirming the existence of a Cu shell in this intermediate product. In contrast, the Cu_2_O_(O)_@Cu_2_O_(P)_@AuPt sample shows the presence of a small amount of Cu^2+^ species, which may be related to slight surface oxidation during the replacement reaction or subsequent exposure to air. Since the binding energies of Cu^+^ and Cu^0^ are very close, it is difficult to unambiguously distinguish them based solely on Cu 2p spectra. Therefore, the Cu LMM Auger spectra were further analyzed, as shown in Figure 2c. Notably, only Cu_2_O@Cu exhibits a distinct feature at around 568 eV, which is characteristic of Cu^0^, further confirming the formation of metallic Cu in this sample. The high-resolution O 1s spectra (Figure S6c,f) can be deconvoluted into two main components, which are assigned to lattice oxygen and adsorbed oxygen species, respectively. Among the three samples, Cu_2_O_(O)_@Cu_2_O_(P)_@AuPt exhibits the highest proportion of lattice oxygen (Figure 2d), suggesting a more ordered surface lattice and a lower concentration of surface oxygen defects. This result implies that the reconstructed outer shell possesses relatively improved surface structural integrity after the galvanic replacement process. The chemical states of noble metals in Cu_2_O_(O)_@Cu_2_O_(P)_@AuPt were further examined as well. As shown in Figure 2e, two peaks located at 84.33 eV and 88.03 eV can be assigned to Au 4f_7/2_ and Au 4f_5/2_ of metallic Au^0^, respectively. In the Pt 4f spectrum (Figure 2f), the peaks centered at around 71.5 eV and 75.0 eV correspond to the spin–orbit doublet of Pt, and the fitting results suggest the coexistence of metallic Pt^0^ and a small amount of oxidized Pt species (Pt^2+^/Pt^4+^). These results collectively demonstrate that AuPt bimetallic nanoparticles were successfully formed on the surface of the Cu_2_O-based heterostructure.

In addition, the elemental composition of the deposited AuPt nanoparticles was further analyzed by EDS spectroscopy. As shown in Figure S7, the atomic percentages of Au and Pt are 1.23% and 0.71%, respectively. The nearly identical atomic contents of Au and Pt confirm an approximately 5:3 atomic ratio between the two elements, providing further evidence for the successful formation of AuPt alloy nanoparticles in the Cu_2_O_(O)_@Cu_2_O_(P)_@AuPt sample.

### 3.2. Photocatalytic Properties

To evaluate the versatility of the as-prepared photocatalysts, TC and MO were selected as model pollutants, which represent antibiotic residues and azo dyes, respectively [45]. Therefore, the use of both TC and MO allows a more comprehensive assessment of photocatalytic applicability toward different organic pollutants.

**Figure 3:**
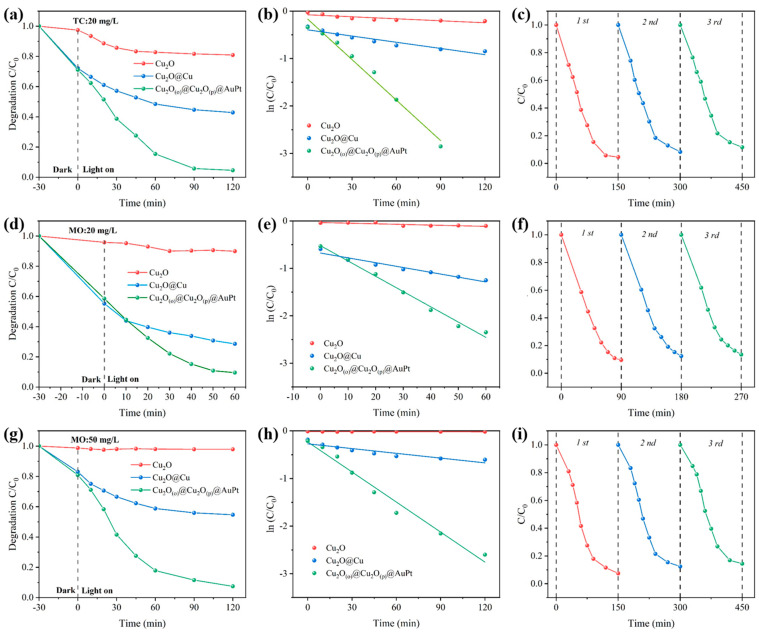
(**a**) Photocatalytic degradation curve of 20 mg/L TC. (**b**) Reaction kinetics fitting diagram. (**c**) Cyclic performance of Cu_2_O_(O)_@Cu_2_O_(P)_@AuPt. (**d**) Photocatalytic degradation curve of 20 mg/L MO. (**e**) Reaction kinetics fitting diagram. (**f**) Cyclic performance of Cu_2_O_(O)_@Cu_2_O_(P)_@AuPt. (**g**) Photocatalytic degradation curve of 50 mg/L MO. (**h**) Reaction kinetics fitting diagram. (**i**) Cyclic performance of Cu_2_O_(O)_@Cu_2_O_(P)_@AuPt.

The photocatalytic degradation performance toward TC (20 mg/L) is shown in Figure 3a–c. Before visible-light irradiation, the suspensions were magnetically stirred in the dark for 30 min to establish adsorption–desorption equilibrium. During this dark period, Cu_2_O exhibited only negligible adsorption toward TC, while Cu_2_O@Cu and Cu_2_O_(O)_@Cu_2_O_(P)_@AuPt showed much more pronounced adsorption, indicating that the reconstructed rough shell is beneficial for pollutant adsorption. Then the light was turned on for 120 min, and the difference in photocatalytic activity became much more evident. Cu_2_O showed only limited TC degradation of 19.1%, and Cu_2_O@Cu displayed a clear improvement to 57.1%. Notably, Cu_2_O_(O)_@Cu_2_O_(P)_@AuPt exhibited the best degradation of 95.4%, corresponding to nearly complete removal of TC under the same conditions. It is worth noting that the competitive adsorption between original pollutants and their intermediate derivatives continuously consumes catalytic active sites, which restricts the continuous oxidation process and prevents 100% removal efficiency. The kinetic plots in Figure 3b further show that the degradation process can be well described by a pseudo-first-order model, and the Cu_2_O_(O)_@Cu_2_O_(P)_@AuPt sample gives the steepest linear slope, confirming its highest apparent reaction rate constant. In addition, the cycling test in Figure 3c demonstrates that this catalyst retains high activity over three consecutive runs, suggesting the outer AuPt alloy shell effectively suppresses photocorrosion of Cu_2_O.

A similar activity trend was also observed for MO (20 mg/L), as shown in Figure 3d–f. Cu_2_O again exhibited very weak photocatalytic activity, whereas Cu_2_O@Cu showed moderate degradation capability. In contrast, Cu_2_O_(O)_@Cu_2_O_(P)_@AuPt achieved the highest degradation efficiency of 90.4%. The corresponding pseudo-first-order kinetic fitting confirms that the final hierarchical heterostructure possesses the fastest degradation rate, and the recycling results indicate that its activity remains high after repeated use.

To further assess the catalyst performance under a higher pollutant loading, the concentration of MO was increased to 50 mg/L. This test is important because the concentration of dye pollutants in practical wastewater is often highly variable, and a higher initial concentration provides a more rigorous evaluation of the tolerance, interfacial charge-transfer capability, and availability of reactive sites. As shown in Figure 3g–i, Cu_2_O_(O)_@Cu_2_O_(P)_@AuPt still exhibited an outstanding photocatalytic degradation efficiency of 92.6% after 120 min, whereas Cu_2_O and Cu_2_O@Cu exhibited degradation efficiencies of 2.1% and 45.3%, respectively. The kinetic and cycling results again confirm the clear superiority and good durability of the Cu_2_O_(O)_@Cu_2_O_(P)_@AuPt. Moreover, this sample was compared with other catalysts reported recently, and the detailed information is shown in Table S2.

Moreover, to further evaluate the structural stability of the photocatalyst after repeated use, the morphology of the recovered Cu_2_O_(O)_@Cu_2_O_(P)_@AuPt sample after the cycling test was examined by SEM. As shown in Figure S8, the used catalyst still maintains a morphology highly similar to that of the fresh sample, without obvious structural collapse or surface deterioration. As shown in Figure S9, the XRD results demonstrate that the recycled sample exhibits nearly identical diffraction peaks compared with the fresh catalyst, indicating that the main crystal structure and phase composition of the composite are well preserved after cyclic reactions without significant phase transformation. Moreover, XPS survey spectra confirm the persistent presence of four characteristic elements (Cu, O, Au, and Pt) on the catalyst surface after recycling. Notably, high-resolution Cu 2p XPS spectra reveal a slight increase in the relative content of Cu^2+^ species after repeated photodegradation reactions. This phenomenon indicates that a minor degree of surface oxidation occurs on the outer Cu_2_O_(P)_ shell during the long-term photocatalytic reaction process, which is a common and negligible surface oxidation behavior for Cu_2_O-based photocatalysts in aqueous photocatalytic environments. The hierarchical particle architecture remains well preserved after repeated photocatalytic reactions, indicating that the catalyst possesses good stability.

### 3.3. Proposed Mechanism

As summarized in Figures S10 and S11, the multilevel core–shell Cu_2_O_(O)_@Cu_2_O_(P)_@AuPt architecture exhibits the highest degradation efficiency, the largest apparent rate constant, and good cycling stability toward both TC and MO. This superior performance originates from the synergistic optimization of surface structure, light harvesting, band alignment, and interfacial charge-transfer behavior.

Adsorption and photodegradation constitute a sequential and integrated process during photocatalysis, so the textural properties of the samples were first examined. As shown in Figure S12 and Table S1, the specific surface areas of Cu_2_O, Cu_2_O@Cu, and Cu_2_O_(O)_@Cu_2_O_(P)_@AuPt are 5.59, 8.89, and 9.26 cm^2^g^−1^, respectively. Compared with octahedral Cu_2_O, the single core–shell Cu_2_O@Cu and the double core–shell Cu_2_O_(O)_@Cu_2_O_(P)_@AuPt increase the specific surface area by approximately 59% and 66%, respectively, which is favorable for enhancing pollutant adsorption and exposing more catalytic active sites. It is also noteworthy that the average pore sizes of the core–shell samples are significantly larger than the pore size of Cu_2_O. Meanwhile, the average pore size of Cu_2_O_(O)_@Cu_2_O_(P)_@AuPt is slightly smaller than that of Cu_2_O@Cu, which can be reasonably attributed to the deposition of AuPt bimetallic nanoparticles on the outer shell. However, another normalized pseudo-first-order kinetics constant (k′) was introduced to evaluate the contribution of surface area to the photocatalytic activity, which represented the ratio of the k value to the surface area [45,46]. As shown in Figure S13, the value of Cu_2_O_(O)_@Cu_2_O_(P)_@AuPt remains markedly higher than that of the other samples, indicating that the enhanced photocatalytic performance is not primarily attributed to the increased surface area.

**Figure 4:**
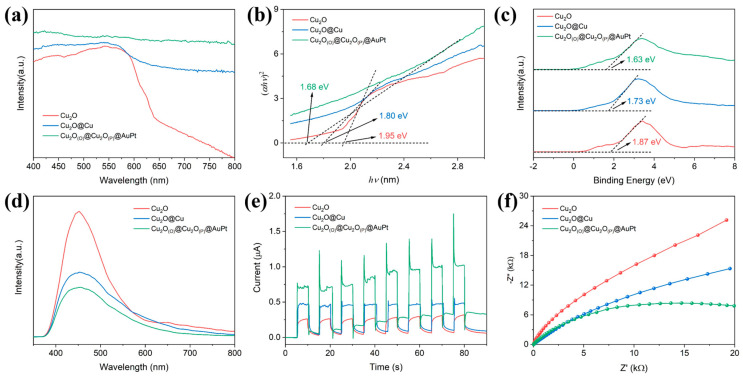
(**a**) UV-vis absorption spectra. (**b**) The corresponding Tauc plots. (**c**) XPS spectra of the valence band. (**d**) PL spectra. (**e**) Photocurrent curves. (**f**) EIS curves.

The enhanced photocatalytic activity is also closely related to the improved optical response. As shown in Figure 4a, all three samples exhibit visible-light absorption, confirming their potential for photocatalytic applications under visible irradiation. However, Cu_2_O shows a pronounced decline in absorption intensity beyond approximately 600 nm, whereas Cu_2_O@Cu and especially Cu_2_O_(O)_@Cu_2_O_(P)_@AuPt maintain much stronger absorption over the whole visible region. This result indicates that the hierarchical double core–shell structure and AuPt loading effectively enhance light harvesting, which is beneficial for generating more photogenerated charge carriers. The band-gap energies were estimated from the Tauc plots shown in Figure 4b. The calculated band gaps of Cu_2_O, Cu_2_O@Cu, and Cu_2_O_(O)_@Cu_2_O_(P)_@AuPt are determined to be 1.93 eV, 1.78 eV, and 1.61 eV, respectively. The progressive narrowing of the band gap demonstrates that structural reconstruction and AuPt deposition modify the electronic structure of Cu_2_O and extend its visible-light response. Among the three samples, Cu_2_O_(O)_@Cu_2_O_(P)_@AuPt possesses the smallest band gap, which is advantageous for absorbing lower-energy visible photons and improving solar-energy utilization.

The valence-band spectra in Figure 4c further reveal changes in the electronic structure after shell evolution and noble-metal incorporation. The valence-band maxima of Cu_2_O, Cu_2_O@Cu, and Cu_2_O_(O)_@Cu_2_O_(P)_@AuPt were measured to be 1.87, 1.81, and 1.63 eV, respectively. The downward shift of the valence-band edge in the hierarchical AuPt-decorated sample suggests a clear modulation of the band structure, which is expected to influence charge migration and surface redox behavior. Combined with the narrowed band gap, the energy band structures of the three samples can be illustrated in Figure S14, which indicates that the double core–shell architecture coupled with AuPt nanoparticles is effective in optimizing the electronic configuration of the photocatalyst.

PL spectroscopy was employed to evaluate the recombination behavior of photogenerated electron–hole pairs. As shown in Figure 4d, the PL intensity decreases in the order of Cu_2_O > Cu_2_O@Cu > Cu_2_O_(O)_@Cu_2_O_(P)_@AuPt. Since stronger PL emission generally corresponds to more severe radiative recombination, the weakest PL signal observed for Cu_2_O_(O)_@Cu_2_O_(P)_@AuPt indicates the most efficient suppression of charge-carrier recombination. This finding confirms that the hierarchical heterointerfaces and AuPt nanoparticles play a critical role in promoting charge separation.

The transient photocurrent responses shown in Figure 4e provide further evidence for improved photoinduced charge dynamics. All samples display stable and reproducible photocurrent signals during repeated on/off irradiation cycles, indicating good photoresponse reversibility. Notably, Cu_2_O_(O)_@Cu_2_O_(P)_@AuPt exhibits the highest photocurrent intensity. EIS was used to probe the interfacial charge-transfer resistance. As shown in Figure 4f, the Nyquist semicircle radius decreases from Cu_2_O to Cu_2_O@Cu and further to Cu_2_O_(O)_@Cu_2_O_(P)_@AuPt. The smallest semicircle observed for the AuPt-decorated double core–shell sample indicates the lowest charge-transfer resistance and the fastest interfacial electron transport. This trend is fully consistent with the PL and photocurrent results.

**Figure 5:**
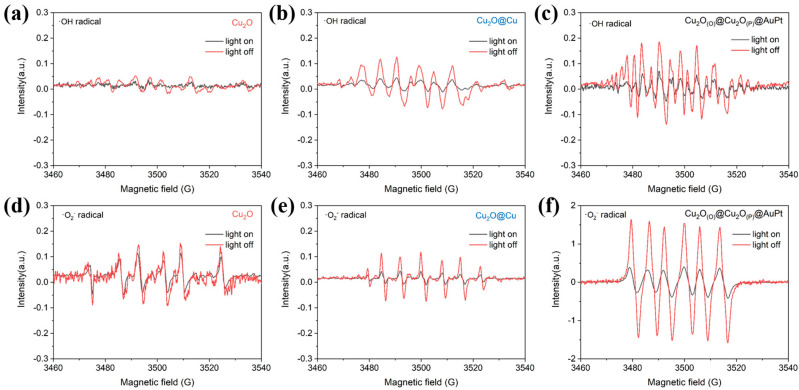
(**a**–**c**) EPR detection of ·OH radicals for Cu_2_O_(O)_@Cu_2_O_(P)_@AuPt. (**d**–**f**) EPR detection of ·O_2_^−^ radicals for Cu_2_O_(O)_@Cu_2_O_(P)_@AuPt.

The EPR spectra in Figure 5 provide direct evidence for the photoinduced formation of reactive oxygen species (ROS) over the as-prepared catalysts. Under visible-light irradiation, the characteristic ·OH and ·O_2_^−^ signals are markedly enhanced relative to those recorded in the dark, confirming that radicals are generated during the photocatalytic process. Among the three samples, Cu_2_O exhibits only weak radical signals, indicating limited ROS production due to rapid recombination of photogenerated carriers. After the introduction of the core–shell structure, Cu_2_O@Cu shows a noticeable increase in both ·OH and ·O_2_^−^ signal intensities, suggesting improved charge separation and surface redox activity. Notably, Cu_2_O_(O)_@Cu_2_O_(P)_@AuPt displays the strongest EPR responses, especially for ·O_2_^−^, demonstrating its highest efficiency for activating dissolved O_2_ and generating oxidative radicals. Therefore, the enhanced photocatalytic performance can be attributed to its superior capability to produce ROS, with ·O_2_^−^ playing the dominant role and ·OH making a secondary contribution in pollutant degradation.

**Figure 6:**
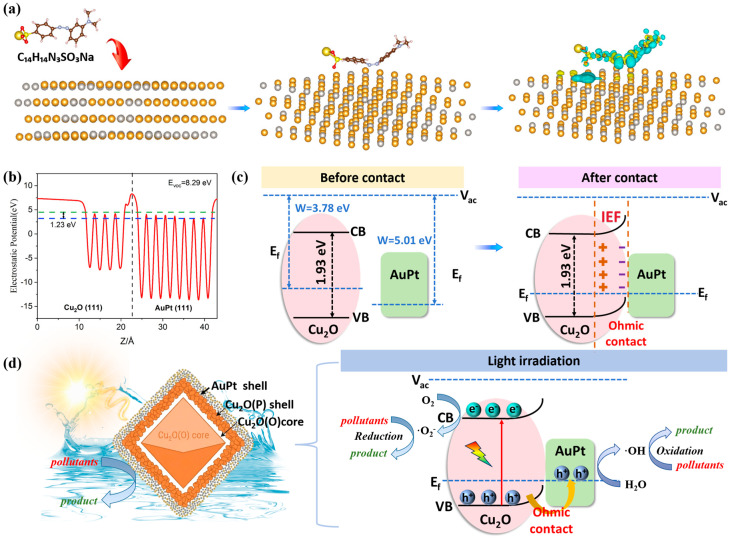
(**a**) Simulation of differential charge density distribution for MO molecule and AuPt interfaces. (**b**) DFT calculations of the work functions of Cu_2_O (111) and AuPt (111). (**c**) Charge-transfer dynamics before contact and after contact. (**d**) Charge-transfer dynamics and carrier separation of Cu_2_O_(O)_@Cu_2_O_(P)_@AuPt under visible light irradiation.

In addition, differential charge density calculations were performed for an adsorption-induced polarized interface in the MO/AuPt adsorption configuration. As shown in Figure 6a, the yellow and cyan regions correspond to electron accumulation and electron depletion, respectively. An obvious interfacial charge redistribution occurs after MO adsorption on the AuPt alloy surface, indicating a strong electronic interaction between the adsorbate and the bimetallic active sites. The charge accumulation and depletion regions are mainly concentrated at the contact interface, confirming that the adsorption of MO on AuPt is not a simple physisorption process but is accompanied by pronounced electron rearrangement and interfacial polarization. Such adsorption-induced charge redistribution is highly favorable for activating the molecular structure of MO and lowering the barrier for subsequent oxidative degradation. More importantly, the simulation reveals significant charge accumulation around the N atoms and the C atoms. The increased electron density around the N atoms suggests that the azo-linked region becomes strongly polarized upon adsorption on the AuPt surface. Since the azo bond (–N=N–) is the key chromophoric group responsible for the color of MO and the integrity of its conjugated electronic structure, this interfacial polarization is expected to strengthen the adsorption of the azo-containing segment on the AuPt alloy and facilitate preferential activation of the azo linkage [47,48]. As a result, cleavage of the azo bond can occur more readily under photocatalytic conditions, leading to rapid decolorization of MO and destruction of the extended π-conjugated system. This result provides a theoretical explanation for the efficient discoloration behavior observed experimentally. As to the azo group, the evident charge accumulation around the aromatic C atoms indicates that the benzene rings are also electronically activated after adsorption. This activation is of considerable mechanistic significance because the aromatic ring in azo dyes is generally chemically stable and difficult to decompose completely. The increased reactivity of these C sites can promote the attack of photoinduced reactive species, thereby facilitating the cleavage of C–C, C–S, and C–N bonds in the molecular skeleton. In particular, the redistribution of charge within the aromatic framework is expected to weaken the delocalized bonding character of the ring and assist in the opening of the benzene ring, which is one of the most kinetically challenging steps in the mineralization of MO. Therefore, the AuPt surface not only accelerates the initial rupture of the azo bond but also promotes the deep oxidation of aromatic intermediates toward complete mineralization.

To clarify the interfacial electronic structure of the Cu_2_O_(O)_@Cu_2_O_(P)_@AuPt, the work functions were further evaluated based on the planar-averaged electrostatic potential. As shown in Figure 6b, the calculated work functions of Cu_2_O (111) and AuPt (111) are 3.78 eV and 5.01 eV, respectively, giving a clear work-function difference of 1.23 eV. This difference provides a strong thermodynamic driving force for interfacial charge redistribution once Cu_2_O and AuPt are brought into intimate contact. As illustrated in Figure 6c, before contact, the Fermi level of Cu_2_O is located closer to the valence band, whereas AuPt possesses a lower Fermi level owing to its larger work function. Upon formation of the Cu_2_O/AuPt heterointerface, electrons tend to transfer from Cu_2_O to AuPt until their Fermi levels reach equilibrium. This interfacial electron migration results in hole accumulation near the Cu_2_O side and electron enrichment on the AuPt side. Consequently, an internal electric field is established at the interface, directed from Cu_2_O to AuPt, which can effectively promote charge separation under light irradiation. The Mott–Schottky plots in Figure S15 confirm the p-type semiconductor of Cu_2_O. When the work function of the metal is higher than that of the p-type semiconductor, hole transport from the semiconductor to the metal is energetically favorable, and the interface generally exhibits ohmic contact characteristics. The type of ohmic contact serves as the core decisive factor among all interfacial effects, which could permit unobstructed cross-interface flow of separated photocarriers and facilitate the extraction of photogenerated holes from p-type Cu_2_O across the interface.

As shown in Figure 6d, this interfacial electronic reconstruction is highly beneficial for photocatalytic charge management. Under visible-light irradiation, Cu_2_O can be excited to generate electron–hole pairs. Driven by the built-in electric field and the favorable interfacial band alignment, photogenerated holes are rapidly transferred to the AuPt cocatalyst, while electrons remain in Cu_2_O or migrate along the semiconductor framework to adsorb O_2_ to produce ·O_2_^−^. As a noble-metal alloy cocatalyst, AuPt not only serves as an efficient charge-transfer mediator but also provides abundant active sites for surface catalytic reactions. As mentioned before, the AuPt alloy can further optimize the adsorption and activation of pollutants, thereby accelerating the overall photocatalytic process.

## 4. Conclusions

In summary, by leveraging the similar reduction potentials of Au and Pt precursors, a multilevel core–shell-structured Cu_2_O_(O)_@Cu_2_O_(P)_@AuPt nanoparticle was synthesized under room-temperature and ambient-pressure conditions. From a photocatalytic perspective, this theoretical result strongly supports a dual function of AuPt in the hierarchical Cu_2_O_(O)_@Cu_2_O_(P)_@AuPt system. On the one hand, AuPt acts as a molecular activation platform by strengthening the adsorption and inducing favorable charge redistribution in key reactive sites of the pollutant molecule. On the other hand, AuPt serves as an efficient charge-transfer mediator through the ohmic contact formed at the Cu_2_O/AuPt interface, which enhances charge separation and promotes the generation of reactive species. The combination of efficient electron extraction, radical generation, and adsorption-induced molecular activation greatly accelerates the stepwise degradation of organic pollutants from chromophore destruction to aromatic ring opening and final conversion into CO_2_ and H_2_O.

## Data Availability

The original contributions presented in this study are included in the article/supplementary material. Further inquiries can be directed to the corresponding authors.

## Supplementary Materials

The following supporting information can be downloaded at https://www.mdpi.com/article/10.3390/ma19143069/s1, Figure S1. (a–c) SEM images of octahedral Cu_2_O particles. (d–f) TEM and HRTEM images of octahedral Cu_2_O particles. (g) SEM-EDS images of octahedral Cu_2_O particles. Figure S2. (a–c) SEM images of Cu_2_O@Cu core-shell particles. (d–f) TEM and HRTEM images of Cu_2_O@Cu core–shell particles. (g) SEM-EDS images of Cu_2_O@Cu core–shell particles. Figure S3. TEM-EDS images of Cu_2_O_(O)_@Cu_2_O_(P)_@AuPt particles. Figure S4. The XRD patterns of the samples. Figure S5. The FTIR spectra of the samples. Figure S6. (a) XPS full spectra of Cu_2_O, (b) Cu 2p fine spectra, (c) O 1s fine spectra, (d) XPS full spectra of Cu_2_O@Cu, (e) Cu 2p fine spectra, and (f) O 1s fine spectra. Figure S7. EDS energy spectrum of the sample: (a) Cu_2_O; (b) Cu_2_O@Cu; (c) Cu_2_O_(O)_@Cu_2_O_(P)_@AuPt. Figure S8. FESEM image of Cu_2_O_(O)_@Cu_2_O_(P)_@AuPt after three photocatalytic tests. Figure S9. (a) XRD patterns, (b) XPS full spectrum, (c) Cu 2p fine spectrum, (d) Au 4f fine spectrum, (e) O 1s fine spectrum, and (f) Pt 4f fine spectrum of Cu_2_O_(O)_@Cu_2_O_(P)_@AuPt after three photocatalytic tests. Figure S10. The corresponding histogram of degradation efficiency. Figure S11. The corresponding kinetic k values of different samples. Figure S12. (a) Nitrogen adsorption–desorption isotherm; (b) the corresponding BJH pore-size distribution. Figure S13. The corresponding kinetic k` values of different samples. Figure S14. Schematic illustration of the band-gap structure alignments. Figure S15. Mott–Schottky plots of Cu_2_O. Table S1. BET of the samples. Table S2. Comparison of degradation of solution with other recently reported catalysts. References [49,50,51,52,53,54,55,56,57,58,59,60,61,62] are cited in the supplementary materials.
