# Supplementary material for: Design of Cu2O(O)@Cu2O(P)@AuPt Multilevel Core–Shell Heterostructures via Mild Reduction Strategy with a Dual Function for Efficient Photocatalytic Degradation
Source: Materials (Basel). 2026 Jul 16;19(14):3069. doi: 10.3390/ma19143069 (PMC13413607; doi:10.3390/ma19143069)
Supplement: Supplementary file 1 [file materials-19-03069-s001.zip › materials-4391177-supplementary.pdf]

Supplementary Data

Design of Cu2O(O)@Cu2O(P)@AuPt Multilevel Core–Shell Heterostructures via Mild Reduction Strategy with a Dual Function for Efficient Photocatalytic Degradation

Bo Ma [1,2,*], Guoqiang Huang [1], Wenwen Hu [1], Wenxue An [1], Gailan Ma [1], Maohui Li [1,3] and Youjun Lu [1,3,*]

1 School of Materials Science & Engineering, North Minzu University, Yinchuan 750021, China
2 Institute of Semiconductor Crystals and Ceramic Materials, Helanshan Laboratory, Yinchuan 750021, China
3 National and Local Joint Engineering Research Center of Advanced Carbon-Based Ceramics Preparation Technology, Yinchuan 750021, China
* Correspondence: boma@nmu.edu.cn (B.M.); 2004006@nun.edu.cn (Y.L.)

Characterization details

The microstructures of samples were investigated by field-emission scanning electron microscopy (FESEM) on a Sigma 500 (Zeiss, Oberkochen, Germany). Transmission electron microscopy (TEM) and high-resolution transmission electron microscopy (HRTEM) analyses were performed on a JEOL JEM-2100 (JEOL Ltd., Tokyo, Japan) transmission electron microscope (TEM) operating at an accelerating voltage of 200 kV. Energy-dispersive spectroscopy (EDS) mapping was carried out with JEOL JEM-F200 (JEOL Ltd., Tokyo, Japan). The phase structures of the products were characterized by an X-ray diffractometer using Shimadzu XRD-6000 (Shimadzu Corporation, Tokyo, Japan) with Cu-Kα radiation (λ=1.54 Å). X-ray photoelectron spectroscopy (XPS) was performed with a Thermo Fisher ESCALAB Xi+ spectrometer (Thermo Fisher Scientific Inc., Delaware, USA) using an Al mono Kα X-ray source (0.05 eV energy step size, 500 μm spot size, and 20.0 eV pass energy). The absorption of UV–visible spectra was studied through a Hitachi U-4100 UV-vis spectrophotometer (Hitachi, Ltd., Tokyo, Japan) using $BaSO_4$ as the reference. The photoluminescence (PL) spectra and fluorescence decay spectra were analyzed by FLS980 (Edinburgh Instruments, Livingston, Scotland, UK) with time-resolved fluorescence spectra. Photo-electrochemical measurements and electrochemical impedance spectra (EIS) were collected from an electrochemical CHI 660E analyzer (CHI Shanghai, Inc., Shanghai, China). The N_2 adsorption–desorption isotherm experiments were performed using an ASAP 2020 Micromeritics analyzer (Micromeritics, Norcross, Georgia, USA), and the surface areas of the as-prepared

samples were determined using the Brunauer–Emmett–Teller (BET) methods. The EPR spectra were analyzed using a Bruker A300 instrument (Bruker, Billerica, Massachusetts, USA), with dimethyl pyrroline N-oxide (DMPO) as the trapping agent.

Computational Simulation

All first-principles calculations were performed using the Vienna Abinitio Simulation Package (VASP) based on density functional theory (DFT). The electron–ion interaction was described by the projector augmented-wave (PAW) method, and the exchange–correlation functional was treated using the generalized gradient approximation (GGA) in the Perdew–Burke–Ernzerhof (PBE) form. The plane-wave cutoff energy was set to 400 eV, and the Brillouin zone was sampled using a Monkhorst–Pack k-point mesh of 9×9×1. During structural optimization, all atoms were fully relaxed until the force on each atom was less than 0.02 $eVÅ^{-1}$ and the total energy convergence criterion reached 1.0×10^{-5} eV. To avoid spurious interactions between periodic slabs, a vacuum layer of 15 Å was introduced along the surface normal direction. The charge-density difference and work function were calculated to elucidate the interfacial charge redistribution and electronic interaction within the heterostructure.

Reaction Chemical Formulas

According to orbital hybridization theory, Cu possesses an outer electronic configuration of $3d^{10}4s^1$, indicating that the single active electron in the 4s orbital can be readily released from the attraction of the nucleus. Therefore, under these

conditions, not all active electrons in Cu nanoparticles would be consumed by Au^{3+} and Pt^{2+} ions. Instead, a small amount of Au^{3+} and Pt^{2+} ions can excite part of the relatively stable electrons in Cu to energy levels near the Fermi level, thereby enhancing the reactivity of Cu nanoparticles, which are denoted as Cu_{Act}.

$$Cu \xrightarrow{Au^{3+}/Pt^{4+}} Cu_{Act} \tag{S1}$$

$$5Cu_{Act} + Au^{3+} + Pt^{2+} \longrightarrow 5Cu^{+} + AuPt \tag{S2}$$

$$2Cu_{Act} + +O_2 + 4H^{+} \longrightarrow Cu_2O + 2H_2O \tag{S3}$$

Equation

$$Degradation\ efficiency = \frac{C_0 - C}{C_0} \times 100\%$$

where C and C_0 are the real-time absorbance acquired at different time intervals and the initial absorbance, respectively.

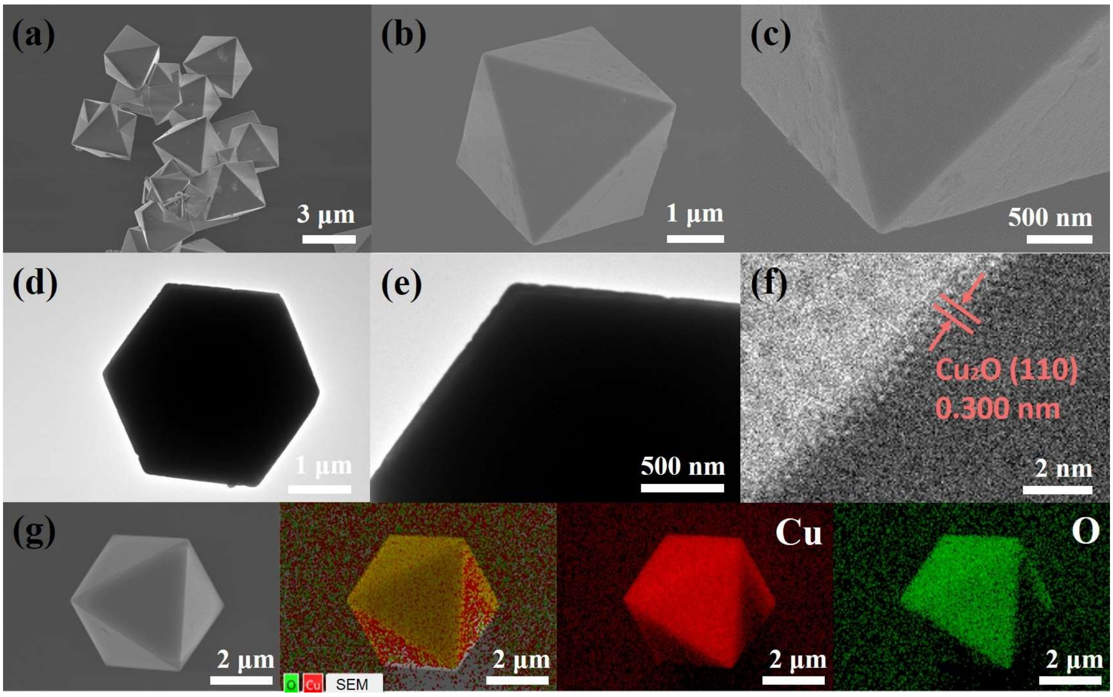

Figure S1. (a-c) SEM images of octahedral Cu_2O particles. (d-f) TEM and HRTEM images of octahedral Cu_2O particles. (g) SEM-EDS images of octahedral Cu_2O particles.

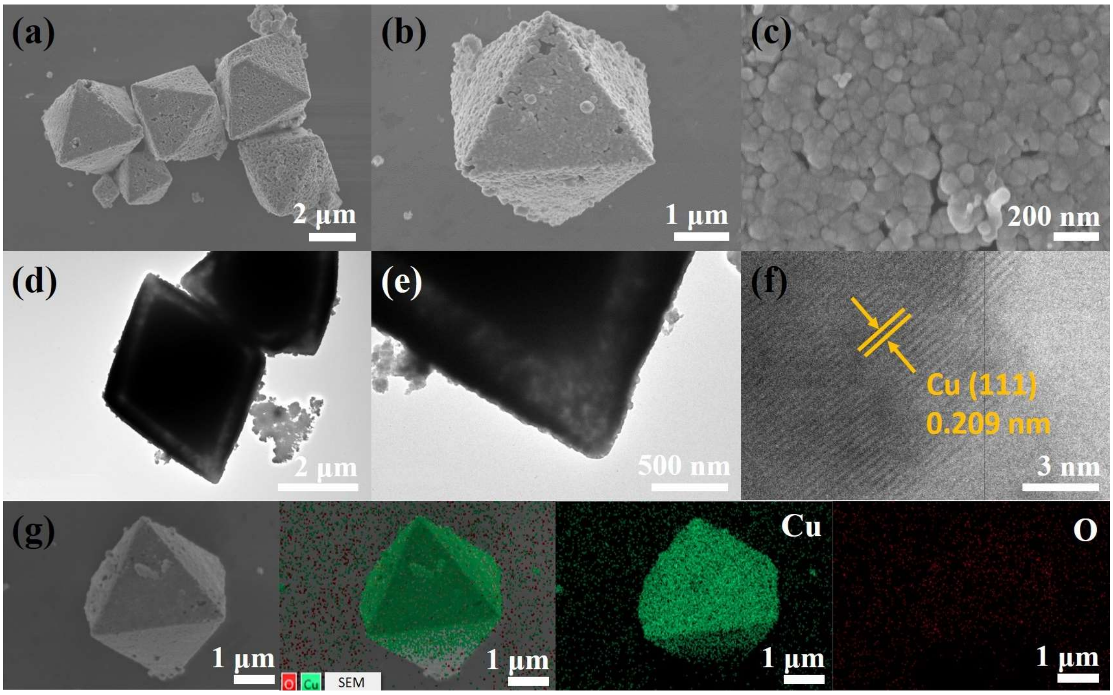

Figure S2. (a-c) SEM images of Cu_2O@Cu core-shell particles. (d-f) TEM and HRTEM images of Cu_2O@Cu core–shell particles. (g) SEM-EDS images of Cu_2O@Cu core–shell particles.

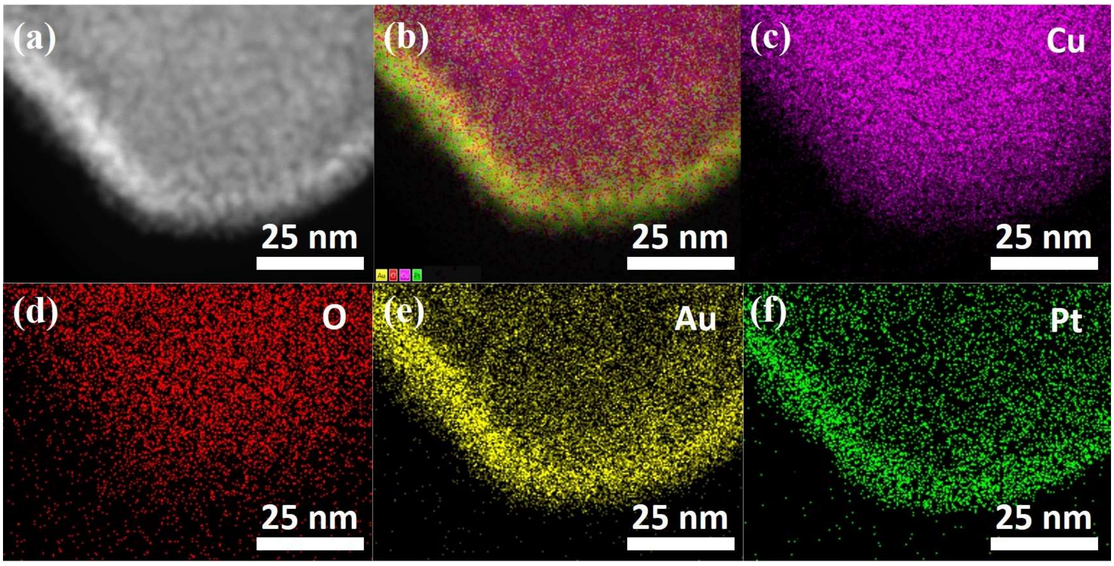

Figure S3. TEM-EDS images of Cu_2O(O)@Cu_2O(P)@AuPt particles.

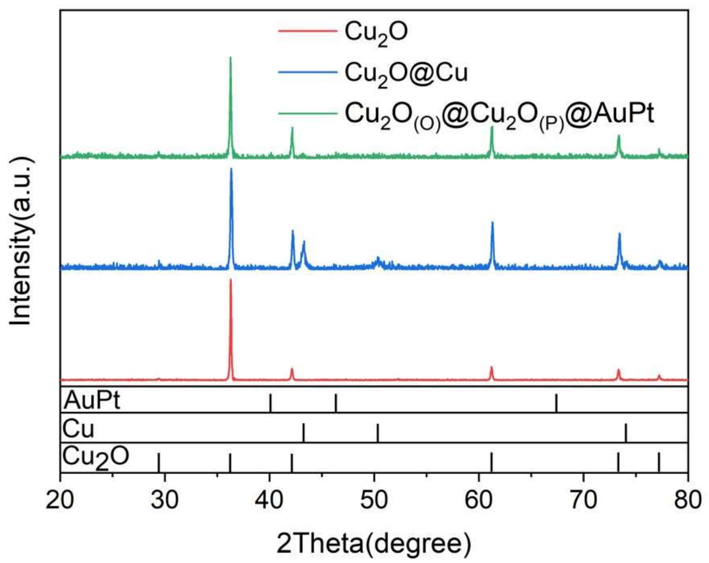

Figure S4. The XRD patterns of the samples.

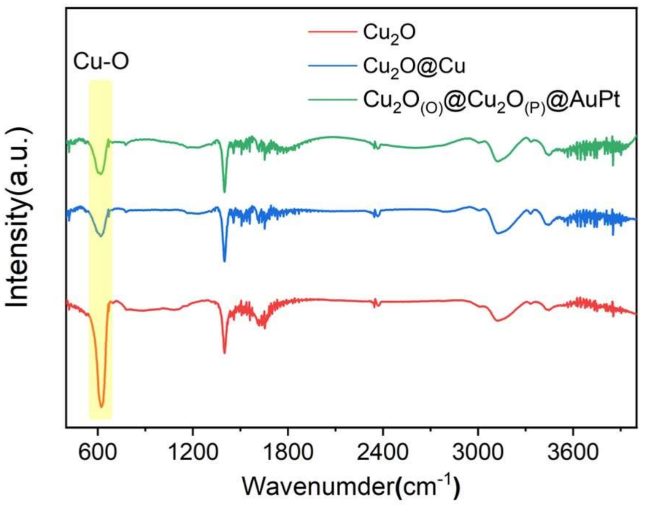

Figure S5. The FTIR spectra of the samples.

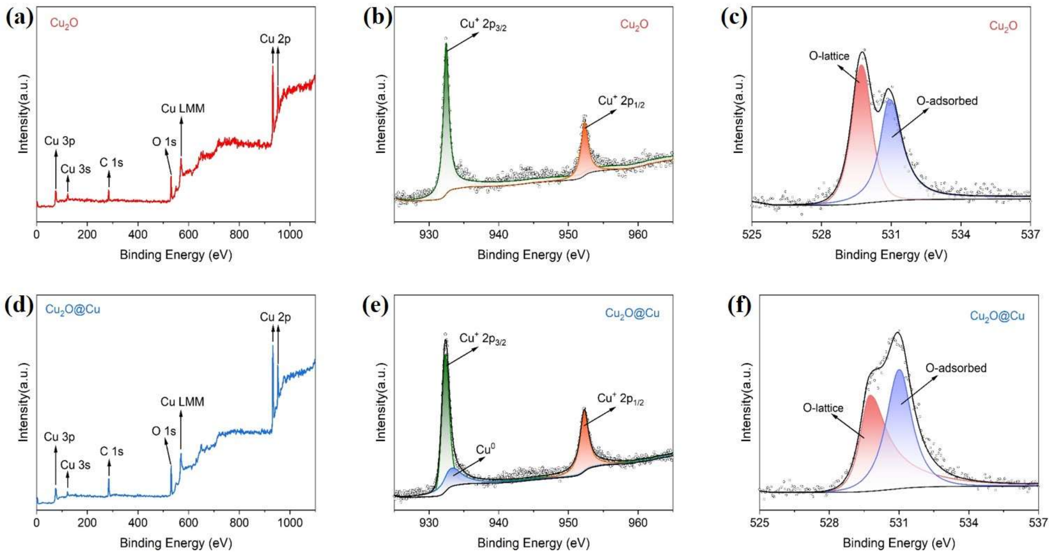

Figure S6. (a) XPS full spectra of Cu_2O, (b) Cu 2p fine spectra, (c) O 1s fine spectra, (d) XPS full spectra of Cu_2O@Cu, (e) Cu 2p fine spectra, and (f) O 1s fine spectra.

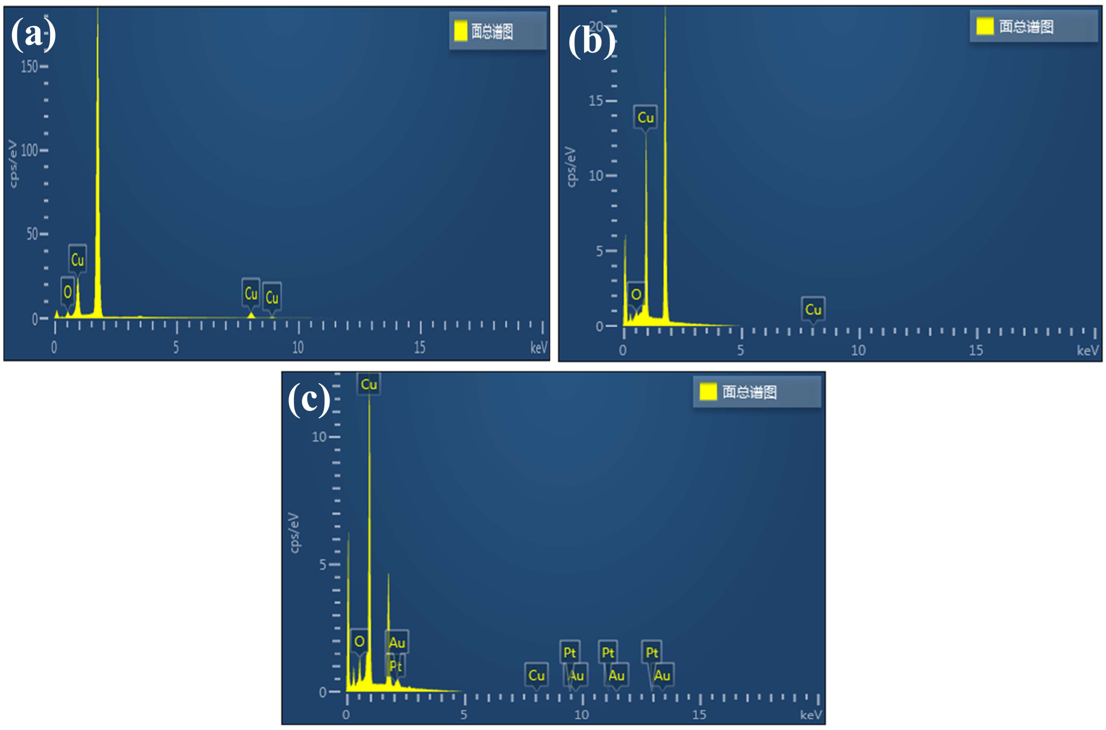

Figure S7. EDS energy spectrum of the sample: (a) Cu_2O; (b) Cu_2O@Cu; (c) $Cu_2O_{(O)}$@$Cu_2O_{(P)}$@AuPt.

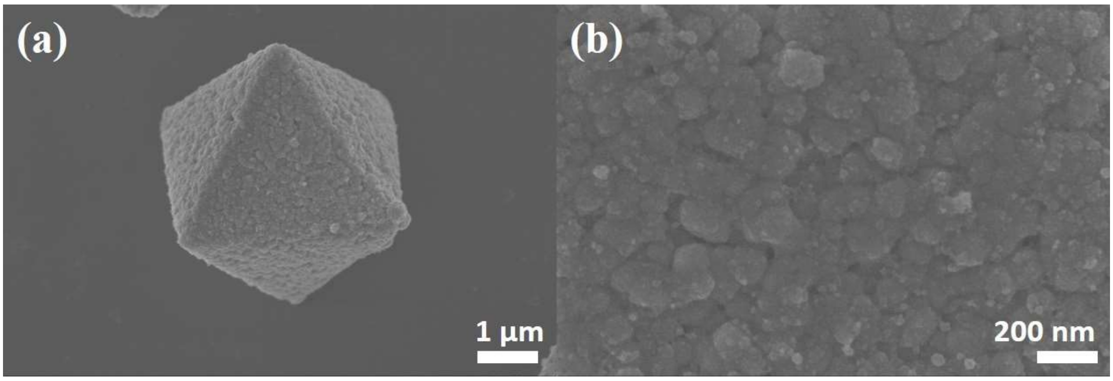

Figure S8. FESEM image of Cu_2O(O)@Cu_2O(P)@AuPt after three photocatalytic tests.

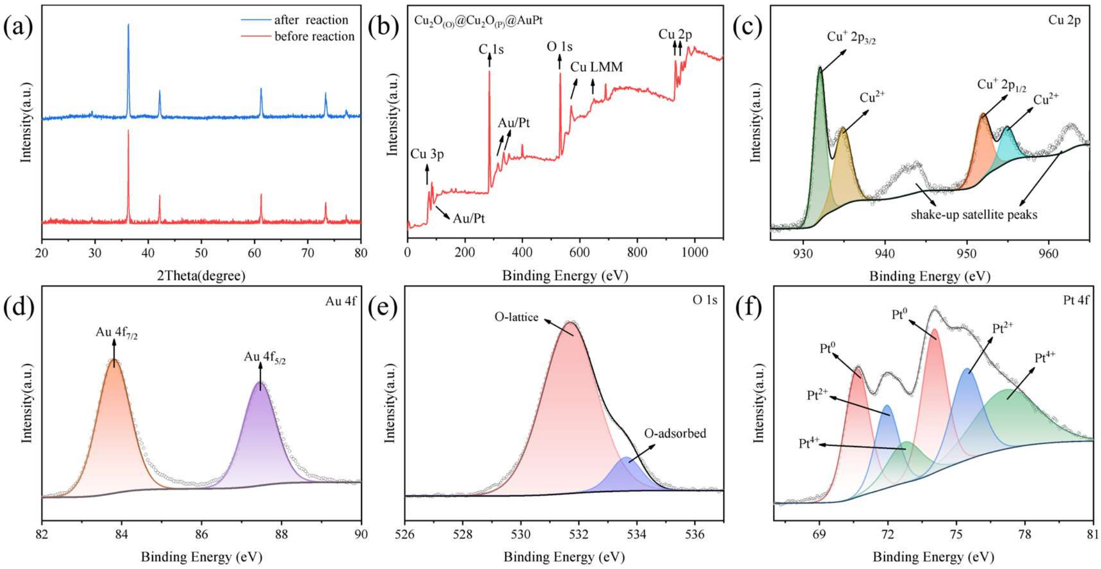

Figure S9. (a) XRD patterns, (b) XPS full spectrum, (c) Cu 2p fine spectrum, (d) Au 4f fine spectrum, (e) O 1s fine spectrum, and (f) Pt 4f fine spectrum of $Cu_2O_{(O)}$@$Cu_2O_{(P)}$@AuPt after three photocatalytic tests.

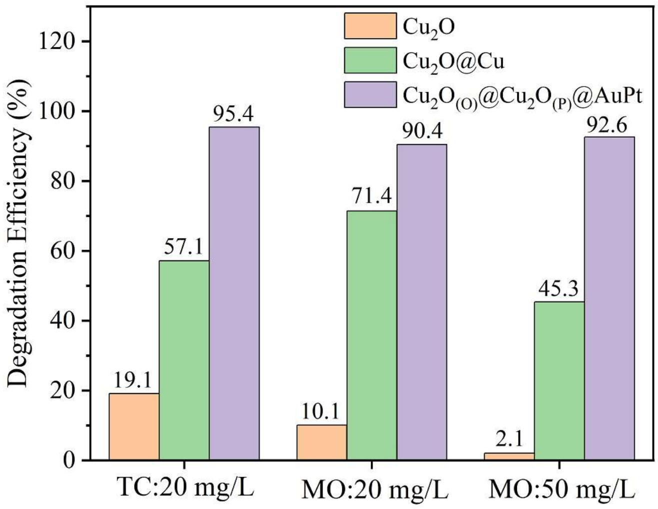

Figure S10. The corresponding histogram of degradation efficiency.

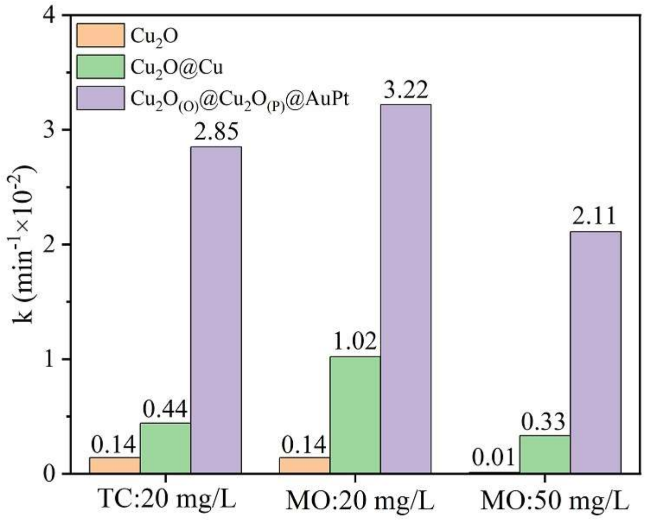

Figure S11. The corresponding kinetic k values of different samples.

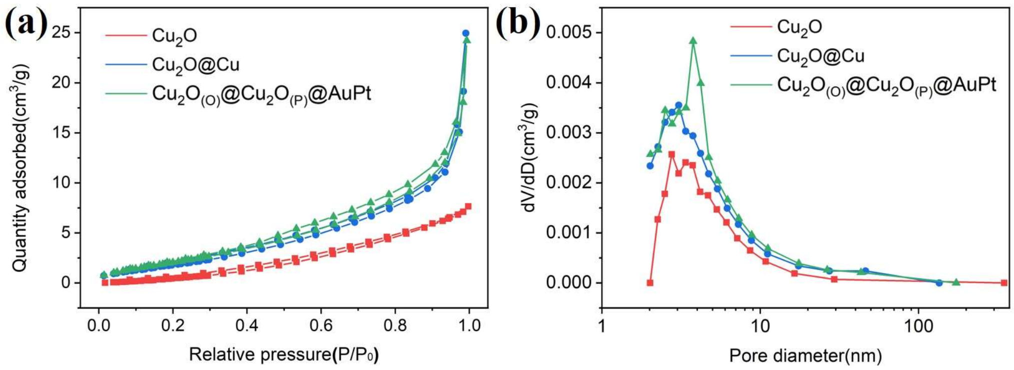

Figure S12. (a) Nitrogen adsorption–desorption isotherm; (b) the corresponding BJH pore-size distribution.

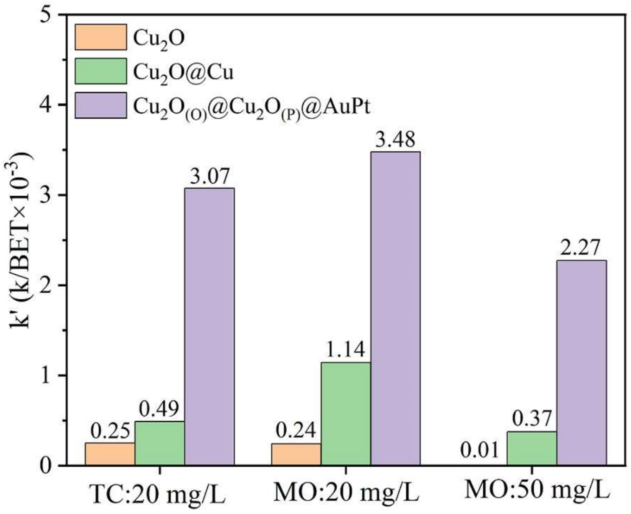

Figure S13. The corresponding kinetic k` values of different samples.

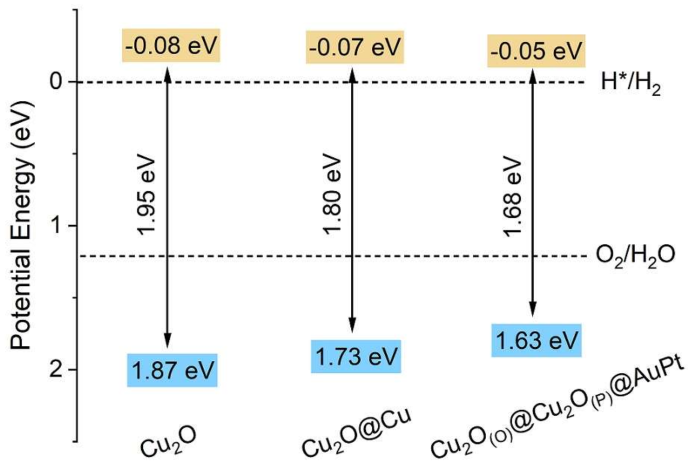

Figure S14. Schematic illustration of the band-gap structure alignments.

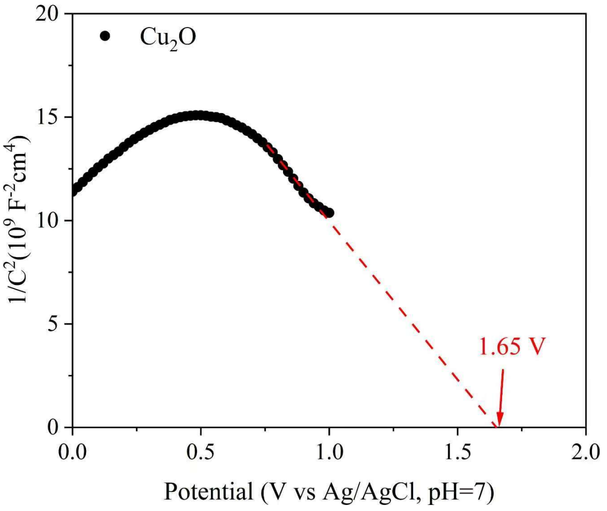

Figure S15. Mott–Schottky plots of Cu_2O.

Table S1. BET of the samples

Description of sample	Specific surface area(cm^2/g)	Total pore volume(cm^2/g)	Average pore size(nm)
Cu_2O	5.59	$1.18*10^{-2}$	8.43
Cu_2O@Cu	8.89	$3.86*10^{-2}$	19.09
$Cu_2O_{(O)}$@$Cu_2O_{(P)}$@AuPt	9.26	$3.74*10^{-2}$	16.16

Table S2 Comparison of degradation of solution with other recently reported catalysts.

Catalysts	Catalyst quantity	Concentration	Light source	Removal capacity (%)(reaction time)	Ref.
$Cu_2O/g\text{-}C_3N_4$-0.2	50 mg	MO 20 mg/L, 100 mL	300 W xenon lamp(λ≥420 nm)	98.3 (90 min)	[1]
Cu_2O NP@ g-C_3N_4 NT	10 mg	MO 10 mg/L, 40 mL	600 W xenon lamp(λ≥420 nm)	96.9 (60 min)	[2]
ZnO/Au@Cu_2O	10 mg	MO 20 mg/L, 40 mL	500 W xenon lamp (simulated sunlight)	93.0 (90 min)	[3]
Cu_2O/SnS_2	30 mg	MO 15 mg/L, 60 mL	300 W xenon lamp	97.0 (75 min)	[4]
Cu_2O/Pt	5 mg	MO 20 mg/L, 100 mL	300 W xenon lamp(λ≥420 nm)	97.0 (60 min)	[5]
g-C_3N_4/FeOOH/Cu_2O	25 mg	MO 50 mg/L, 50 mL	500 W xenon lamp(λ≥400 nm)	93.5 (90 min)	[6]
$Cu_2O/g\text{-}C_3N_4$/Ag	50 mg	MO 20 mg/L, 100 mL	300 W xenon lamp(λ≥420 nm)	98.1 (45 min)	[7]
MBC@Cu_2O/Ag	20 mg	MO 50 mg/L, 50 mL	300 W xenon lamp(λ≥420 nm)	97.8 (60 min)	[8]
Cu_2O/Cu	5 mg	MO 25 mg/L, 50 mL	300 W xenon lamp	89.0 (60 min)	[9]
Cu_2O/Ti-Fe LDH	20 mg	TC 40 mg/L, 50 mL	300 W xenon lamp(λ≥420 nm)	82.5 (60 min)	[10]
Cu_2O/Cu_7S_4	10 mg	TC 20 mg/L, 50 mL	300 W xenon lamp(λ≥400 nm)	95.9 (60 min)	[11]
Cu_2O/BiOCl	40 mg	TC 20 mg/L, 100 mL	300 W Xenon lamp(λ≥400 nm)	90.3 (80 min)	[12]
ZnO/Cu_2O	10 mg	TC 20 mg/L, 50 mL	300 W xenon lamp(λ≥420 nm)	95.3 (120 min)	[13]
Ag/$Cu_2O/BiVO_4$	40 mg	TC 20 mg/L, 100 mL	500 W xenon lamp(AM 1.5)	89.4 (30 min)	[14]
Cu@Cu_2O/CuO	20 mg	TC 50 mg/L, 100 mL	300 W xenon lamp(λ≥420 nm)	99.4 (20 min)	[15]
$Cu_2O_{(O)}$@$Cu_2O_{(P)}$ @AuPt	10 mg	MO 20 mg/L, 50 mL	300 W xenon lamp(λ≥420 nm)	90.4 (60 min)	This work
$Cu_2O_{(O)}$@$Cu_2O_{(P)}$ @AuPt	10 mg	MO 50 mg/L. 50 mL	300 W xenon lamp(λ≥420 nm)	92.6 (120 min)	This work
$Cu_2O_{(O)}$@$Cu_2O_{(P)}$ @AuPt	10 mg	TC 20 mg/L, 50 mL	300 W xenon lamp(λ≥420 nm)	95.4 (120 min)	This work

References:

1. Yang, S.; Li, K.; Huang, P.; Liu, K.; Li, W.; Zhuo, Y.; Yang, Z.; Han, D. Dual-functional Cu_2O/g-C_3N_4 heterojunctions: a high-performance SERS sensor and photocatalytic self-cleaning system for water pollution detection and remediation. *Microsyst. Nanoeng* **2024**, *10*, 198.
2. Wang, Z.; Wang, J.; Iqbal, W.; Yang, L.; Shi, M.; Chang, N.; Qin, C. Controllable fabrication and enhanced photocatalysis of Cu_2O NP@g-C_3N_4 NT composite on visible-light-driven degradation of organic dyes in water. *Mater. Today Sustain* **2022**, *20*, 100239.
3. Yuan, X.; Pei, F.; Luo, X.; Hu, H.; Qian, H.; Wen, P.; Miao, K.; Guo, S.; Wang, W.; Feng, G. Fabrication of ZnO/Au@Cu_2O heterojunction towards deeply oxidative photodegradation of organic dyes. *Sep. Purif. Technol* **2021**, *262*, 118301.
4. Wang, H.; Pan, Y.; Yang, J.; Tang, B.; Ye, F.; Gao, C.; Han, C.; Yu, C. Interfacial charge transfer dynamics in S-scheme Cu_2O/SnS_2 heterojunction for enhanced photocatalytic degradation of organic pollutants. *Appl. Surf. Sci* **2026**, *719*, 165030.
5. Ma, B.; Ma, G.; Xu, Q.; Huang, G.; Shen, H.; Li, M.; Li, D.; Feng, G.; Wang, Y.; Lu, Y. Fabrication of plasmonic Hollow-Cu_2O/Pt nanocages with inter-embedded nanoparticles subunits heterojunctions for efficient photocatalytic degradation and hydrogen evolution. *Appl. Surf. Sci* **2025**, *690*, 162656.
6. Zou, X.; Zhang, X.n.; Wang, Y.; Gu, F.; Zhang, X. Facile construction of g-C_3N_4/FeOOH/Cu_2O ternary heterojunction with enhanced visible light photocatalytic performance. *J. Alloys Compd* **2025**, *1046*, 184842.
7. Li, K.; Li, W.; Huang, P.; Zhuo, Y.; Yang, Z.; Wang, B.; Liu, R.; Liu, B.; Hou, E.; Yang, S.; et al. Z-scheme Cu_2O/g-C_3N_4/Ag heterostructures: A novel approach for simultaneous water pollutant removal and real-time SERS monitoring. *J. Environ. Chem. Eng* **2025**, *13*, 119217.
8. Zhang, Y.; Chen, J.; Wang, Y.; Dou, H.; Lin, Z.; Gao, X.; Chen, X.; Guo, M. Cu_2O/Ag-coated wood-based biochar composites for efficient adsorption/photocatalysis synergistic degradation of high-concentration azo dyes. *Appl. Surf. Sci* **2024**, *647*, 158985.
9. Li, J.; Huang, L.; Yang, Z.; Liu, Z.; Sun, X. Construction of Cu_2O/Cu heterojunction with hierarchical hollow sphere structure as visible-light driven photocatalyst for efficient water remediation. *J. Environ. Chem. Eng* **2022**, *10*, 108020.
10. Ye, L.; Sun, S.; Yang, X.; Chen, X.; Yang, B.; Yun, D.; Yu, X.; Yang, M.; Yang, Q.; Liang, S.; et al. Mechanism insight into the enhanced photocatalytic purification of antibiotic through encapsulated architectures coupling of crystalline Cu_2O/amorphous TiFe layer double hydroxide. *J. Mater. Sci. Technol* **2023**, *167*, 161-170.
11. Yuan, X.; Huang, Z.; Li, J.; Meng, Y.; Gu, Z.; Xie, B.; Ni, Z.; Xia, S. The S-Cu-O bonds boosted efficient photocatalytic degradation of semi-coherent interface Cu_2O/Cu_7S_4 heterojunction. *Sep. Purif. Technol* **2023**, *306*, 122689.

12. Yuan, X.; Yang, J.; Yao, Y.; Shen, H.; Meng, Y.; Xie, B.; Ni, Z.; Xia, S. Preparation, characterization and photodegradation mechanism of 0D/2D Cu_2O/BiOCl S-scheme heterojunction for efficient photodegradation of tetracycline. *Sep. Purif. Technol* **2022**, *291*, 120965.
13. Cui, J.; Ye, L.; Chen, X.; Li, J.; Yang, B.; Yang, M.; Yang, Q.; Yun, D.; Sun, S. Simultaneously promoting adsorption and charge separation in Z-scheme ZnO/Cu_2O heterojunctions for efficient removal of tetracycline. *Appl. Surf. Sci* **2023**, *638*, 158046.
14. Ding, J.; Wang, J.; Li, Y.; Wang, R.; Luo, J.; Zhang, Y.; Zhang, J.Z.; Sun, Y. Construction of Ag/Cu_2O/$BiVO_4$ p-n heterojunction photocatalyst for highly efficient antibiotic removal. *J. Alloys Compd* **2026**, *1068*, 188466.
15. Yang, Z.; Liu, H.; Yang, X.; Zhang, A.; Sa, S.; Wang, Z. Plasma-Synthesized MOF-Derived Cu@Cu_2O/CuO Photocatalysts for Rapid Tetracycline Degradation. *ACS Sustainable Chem. Eng* **2026**, *14*, 6515-6527.
